# Energy-sparing effect of oregano (*Origanum vulgare* subsp. *hirtum*) essential oil and its mixtures in broilers: Improved feed efficiency via optimized intestinal health

**DOI:** 10.1016/j.psj.2025.106341

**Published:** 2025-12-29

**Authors:** Xiaotong Li, Sihuan Wang, Libo Zhang, Desheng Li, Huiying Li, Qiyue Zhang, Changmin Jin, Jiali Wang, Jun Wang, Jie Han, Fengxia Liu, Shunshun Jin, Lizhi Jin, Donghui Shi

**Affiliations:** a College of Animal Husbandry and Veterinary Medicine, Jinzhou Medical University, Jinzhou, 121001, China; b Liaoning Provincial Key Laboratory of Animal Product Quality and Safety, Jinzhou, 121001, China; c Fuxin Mongolian Autonomous County Modern Agricultural Development Service Center, 123100, China; d Huludao City Agricultural Comprehensive Administrative Law Enforcement Team, Huludao, 125000, China; e Guangzhou Meritech Bioengineering Co. Ltd., Guangzhou, 510300, China

**Keywords:** Oregano essential oil and mixtures, Growth performance, Intestinal health, Broiler

## Abstract

The study aimed to investigate the energy-saving effects of oregano essential oil and its compounds in low-metabolizable-energy diets for broilers. A total of 600 one-day-old Arbor Acres broilers of similar body weight were randomly assigned to five dietary treatments, each consisting of six replicates with 20 birds per replicate, over a 42-day trial period. The control group (Ctrl) received a basal diet, whereas the low-energy group (LC) was fed a diet with ME reduced by 210 kJ/kg relative to the Ctrl group. Three supplemented groups were formulated based on the LC diet: LCO (LC + 200 mg/kg oregano essential oil, OEO), LCT (LC + 200 mg/kg oregano-clove essential oil composite, OEC), and LCM (LC + 1000 mg/kg oregano essential oil-lauric acid composite, OEL). Results revealed that reducing dietary metabolizable energy (ME) increased the feed-to-gain ratio (F/G) throughout the trial period. However, supplementation with essential oils on the low-energy diet significantly decreased F/G, enhanced antioxidant enzyme activities, increased secretory immunoglobulin A (SIgA) levels in the duodenum and ileum, and reduced inflammatory cytokine levels in the intestinal mucosa (*P* < 0.05). Morphologically, essential oils improved intestinal structure by increasing villus height, decreasing crypt depth, and elevating the villus-height-to-crypt-depth ratio (VH:CD). Furthermore, OEC and OEL treatments promoted intestinal health by increasing fecal *Lactobacillus* counts while reducing *Escherichia coli* and *Salmonella* counts (*P* < 0.05). 16S rRNA analysis further demonstrated that OEC and OEL enhanced microbial diversity and modulated the composition of the cecal microbiota. In summary, oregano essential oil and its compounds can mitigate the adverse effects of low-metabolizable-energy diets by promoting intestinal development, modulating gut microbiota, and enhancing antioxidant and immune functions. The oregano essential oil-lauric acid complex demonstrated particularly notable effects, providing new insights for developing energy-saving feed additives.

## Introduction

Over the past few decades, aromatic plants have gained attention as sustainable and cost-effective feed additives, offering various bioactive properties that enhance animal health and production performance (Christaki et al., 2020; Giannenas et al., 2018). Among these, oregano essential oil (OEO), extracted from *Origanum vulgare L.*, has demonstrated broad-spectrum antimicrobial, antioxidant, and immunomodulatory effects, and contributes to improved gut microbiota balance in broilers (Zhang et al., 2021a), and shows significant synergistic antibacterial properties (Hu et al., 2023), immune function (Felici et al., 2023) and intestinal health (Zhao et al., 2023) with various compounds in vitro and vivo (Sidiropoulou et al., 2020).

Medium-chain fatty acids (MCFAs), such as lauric acid (LA), are rapidly absorbed via passive diffusion and serve as an immediate energy source for intestinal epithelial cells (Çenesiz and Çiftci, 2020). They enhance immune function, reduce mortality, and exhibit antioxidant properties in poultry (Sengupta et al., 2015). Furthermore, LA supports intestinal development, improves nutrient absorption, and consequently enhances growth performance (Wu et al., 2021b). Similarly, clove essential oil (CEO) extracted mainly from the leaves of *Syzygium aromaticum* has been reported to exhibit antibacterial and antifungal (Hu et al., 2018), antitoxin (Afrendi et al., 2023), anti-inflammatory (Nikoui et al., 2017), antioxidant activities (Fauzya et al., 2019). Nevertheless, the biological effects of dietary supplementation with a combination of oregano essential oil and clove essential oil in broilers remain poorly understood.

Dietary energy supply is a key regulator of growth, metabolism, and physiological homeostasis in broilers (Inci et al., 2016; Sakomura, 2004). The rising cost and intensifying competition for feed resources have driven the industry to explore strategies for maintaining production performance while reducing dietary energy levels (Prates, 2025). However, directly lowering metabolizable energy (ME) to conserve resources often disrupts the body’s energy balance, leading to compensatory growth suppression and reduced feed efficiency (Lara and Rostagno, 2013). Thus, maintaining high efficiency under energy restriction has become a central focus in nutritional research. This study proposes the supplementation of oregano essential oil into low-ME diets. By improving gut barrier function, modulating microbiota, and enhancing nutrient absorption, it aims to provide physiological compensation through the dimension of improving energy utilization efficiency, thereby offering a novel approach for developing green feed additives in support of precision nutrition and sustainable poultry production.

## Materials and methods

### Ethics and procedures

The experiment was conducted in compliance with the guidelines of the Chinese Ethics Committee for the Protection and Use of Agricultural Animals and with relevant guidelines and regulations (AW02211202-1-1, Beijing, China). All experimental protocols which include encompassing husbandry, euthanasia, experimental procedures, and biosecurity precautions, adhered to the Guidelines for Experimental Animals of the Ministry of Science and Technology (Beijing, China).

### Chemicals and reagents

All additive supplementations were provided by Meritech Bioengineering Co. Ltd., Guangzhou, China. The OEO was in the form of a powder, which contains 5% essential oil extracted from *Origanum vulgare* subsp. *hirtum* plants and 95% natural feed-grade inert carrier. OEC or OEL is the mixture of 8% Origanum vulgare subsp. hirtum essential oil with 5% commercial clove oil or 20% lauric acid, respectively. The detailed chemical components of OEO are presented in the previous study (Cheng et al., 2017), all additives were uniformly incorporated into the feed using a stepwise premixing process.

### Birds, dietary treatments, and experimental design

A total of 600 one-day-old Arbor Acres broilers of mixed sex and similar body weight were randomly assigned to five dietary groups (six replicates of 20 birds each). All birds were vaccinated against Marek’s disease, infectious bronchitis, and Newcastle disease. From days 0 to 11, all groups received a common maize-soybean starter diet, followed by phase-specific grower (days 12 −30) and finisher (days 31 −42) diets. The diet of the negative control group (Ctrl) was formulated to meet the nutrient requirements of broilers recommended by the National Research Council as shown in Table 1. The low-energy control group (LC) received the same starter diet as the normal-energy control (Ctrl) from days 0 to 11. Thereafter, LC was fed grower (days 12 −30) and finisher (days 31 −42) diets formulated with reduced apparent metabolizable energy (AME). The AME reduction was achieved by slightly lowering the soybean oil inclusion: soybean oil was decreased from 4.50% to 4.49% in the grower diet and from 5.00% to 4.99% in the finisher diet, with the 0.01% shortfall in each phase replaced by zeolite powder. Consequently, the resulting AME of the grower and finisher diets for LC was 210 kJ/kg lower than that of the corresponding Ctrl diets. The LCO, LCT, and LCM groups were fed the LC diet supplemented with OEO (200 mg/kg), OEC (200 mg/kg), or OEL (1000 mg/kg), respectively.

**Table 1:** Composition and nutrient levels of basal diets (air-dry basis) (as %).

Items	Starter (1-11 d)	Grower (12-30 d)	Finisher (31-42 d)
Ingredients			
Corn	58.01	58.47	59.44
Peeling soybean meal	34.00	10.00	0
Soybean meal	0	17.00	25.00
Corn gluten meal	1.50	4.50	5.00
Soybean oil	2.40	4.50	5.00
CaHPO_4_	1.10	1.30	1.30
Zeolite powder	1.10	1.20	1.20
NaCl	0.28	0.28	0.28
NaHCO_3_	0.10	0.10	0.10
Glutamic acid residue	0	1.00	1.00
Sodium humate	0	0.10	0.10
L-Lysine Sulfate (98%)	0.53	0.65	0.70
DL-Methionine (99%)	0.25	0.20	0.18
L-Threonine (98.5%)	0.13	0.10	0.10
Choline chloride (50%)	0.10	0.10	0.10
Vitamin and mineral Premix1*	0.50	0.50	0.50
Total	100.00	100.00	100.00
Nutrient levels2⁎			
ME/(MJ/Kg)	12.32	13.57	13.73
Crude protein	21.97	20.11	19.98
Calcium	0.87	0.85	0.84
Available phosphorus	0.43	0.42	0.38
L-Lysine	1.52	1.37	1.34
DL-Methionine	0.58	0.57	0.55
Methionine + Cysteine	0.90	0.85	0.83
L- Threonine	0.95	0.76	0.79

1* The premix provided the following per kg of diets: VA l0 000 IU, VD_3_ 2750 IU, VE 50 IU, VK_3_ 3 mg, VB_1_ 2 mg, VB_2_ 6 mg, VB_6_ 3 mg, d-calcium pantothenate 12 mg, VB_l2_ 2.03 mg, niacin 26 mg, biotin 0.15 mg, folic acid 1.5 mg, Zn 75 mg, Mn l20mg, Fe 100 mg, Cu 15 mg, Se 0.3 mg, I 1 mg.

2⁎ Asterisked values for amino acids, available phosphorus and metabolizable energy are calculated; all others are analytically determined.

Birds were housed in separate cages at a commercial farm in China under controlled conditions. The temperature was maintained at 33-34 °C for the first week, then gradually reduced by 3 °C per week until reaching 24 °C, and kept constant thereafter. Continuous lighting was provided. Health status was monitored twice daily, and feed and water were available ad libitum throughout the 42-day trial.

### Sample collection and processing

On day 42 of the trial, a total of 30 hens with similar body weights (six birds per group, with one broiler randomly selected from each replicate) were euthanized at a local slaughterhouse following a 12-hour fast. Immediately after euthanasia, the birds were dissected, and the jejunum and ileum were carefully excised for further processing. For intestinal morphology assessment, 1-cm segments from the midpoint of the jejunum and ileum were fixed in 10% neutral-buffered formalin. Concurrently, 2 cm tissue sections from the same locations, free of digesta, were flushed with physiological saline followed by cold phosphate-buffered saline (PBS, pH 7.2). These tissue samples were then homogenized at 4°C, and the supernatant was collected and stored. Approximately 0.5 g of mucosal epithelium from both the jejunum and ileum was scraped with a sterile scraper, collected into 2 mL cryotubes, and snap-frozen in liquid nitrogen. For microbial analysis, cecal digesta were collected into 5 mL cryotubes, immediately snap-frozen in liquid nitrogen, and stored at −80 °C until further use. All samples were ultimately stored at −80 °C.

### Growth performance

The body weight and feed intake were measured per cage on days 1, 21, and 42. Mortality was recorded daily from day 0 to day 42. The average daily gain (ADG) and the feed-to gain ratio (F/G), adjusted for mortality, were calculated for the periods of days 1-21, 21-42, and 1-42.

### Intestinal immunity and antioxidant capacity

The activities of total antioxidant capacity (T-AOC; No. A015-2-1), catalase, glutathione peroxidase (GSH-Px; No. A005-1-2), superoxide dismutase (SOD; No. A001-3-2), and malondialdehyde (MDA; No. A003-1-2), as well as Immunoglobulins A, M, and G (IgA, IgM, and IgG; Nos. H108-1-2, H109-1-2, and H106-1-1) in the mucosal epithelial tissues of the jejunum and ileum, were measured using an automatic biochemical analyzer (Hitachi 7600, Hitachi High-Technologies Corporation, Tokyo, Japan). Additionally, secretory immunoglobulin A (SIgA; No. H108-2-2)was assessed, following the instructions provided by commercial ELISA kits (Nanjing Jiengcheng Bioengineering Institute, China).

### Intestinal morphological measurements

Tissue samples from the jejunum and ileum, fixed in 10% neutral-buffered formalin, were processed through dehydration, paraffin embedding, and sectioning at 3 μm thickness. Sections were then stained with hematoxylin and eosin (H&E). Histological examination was performed using an Olympus IX51 inverted microscope equipped with a Microcomp integrated digital imaging analysis system (version 6.0, Olympus Optical Co., Ltd., Tokyo, Japan). For morphometric analysis, images were captured under a 4 × EPlan objective (providing 40 × total magnification). Villus height (VH) and crypt depth (CD) were measured for 10 intact, well-oriented villi and their corresponding crypts per intestinal section per bird. Mean VH and CD were calculated for each individual bird, and the villus height-to-crypt depth ratio (VH:CD) was subsequently determined.

### Determination of fecal microbiota

On days 21 and 42, fresh fecal samples (1 g) were aseptically collected from each bird per replicate into sterile bags for microbiological analysis. For bacterial enumeration, each sample was immediately homogenized in a ten-fold volume of sterile 0.9% NaCl solution (10% w/v), followed by serial decimal dilutions from 10⁻¹ to 10⁻⁷. *Lactobacillus spp., Salmonella spp.,* and *Escherichia coli* were enumerated using Rogosa agar, Shigella agar, and MacConkey agar, respectively. Plates for *Lactobacillus* was incubated anaerobically at 37°C for 48 hours within sealed anaerobic jars, while those for *Escherichia coli* and *Salmonella* were incubated aerobically at 37°C for 24 h. Bacterial counts were recorded and expressed as log₁₀ colony-forming units per gram of feces (log₁₀ CFU/g).

### Immuno-related genes expression analysis by RT-qPCR

Gene expressions (IL-2, IL-4, IL-6, IL-10, IL-1β, TNF-α, IFN-γ) were measured by RT-qPCR. Total RNA was extracted from the mucosa of the jejunum and ileum using the YuBioLab TransZol Up Plus RNA Kit (Catalog Number: ET111-01, TransGen Biotech Co., Ltd., Beijing, China). Quantitative PCR was then conducted on a QuantStudio 3 Real-Time PCR System (Applied Biosystems, Thermo Fisher Scientific). Total RNA was reverse-transcribed using the EasyScript® One-Step gDNA Removal and cDNA Synthesis SuperMix (Catalog Number: AET311-02, TransGen Biotech Co., Ltd., Beijing, China). The resulting cDNA was utilized for RT-qPCR on an Applied Biosystems instrument to assess gene expression, following the standard protoco l indicated by the 2^-ΔΔCT^ method (Livak and Schmittgen, 2001). The RT-qPCR reaction was performed in a 20-μL mixture containing 10 μL of 2 × PerfectStart Green qPCR SuperMix (TransGen Biotech), 0.4 μL of each forward and reverse primer, 6 μL of cDNA template, and 3.2 μL of nuclease-free water. The thermal cycling conditions were as follows: initial denaturation at 94 °C for 30 s; followed by 40 cycles of denaturation at 94 °C for 5 s, annealing at 60 °C for 15 s, and extension at 72 °C for 10 s. A melting curve analysis was performed from 65 °C to 95 °C to verify amplification specificity. All reactions were run in triplicate, and β-actin was used as the reference gene for normalization. The primer sequences specific to birds were synthesized by Sangon Biotech Co., Ltd. (Shanghai, China) and are presented in Table 2.

**Table 2:** Primer Sequences for RT-qPCR.

Target gene	Primer sequences (5′→3′)	Gene bank No.	Product size(bp)
β-Actin	F: GATTTCGAGCAGGAGATGGC R: GCCAATGGTGATGACCTGAC	NM_205518.2	90
IL2	F: TCGAGCTCTACACACCAACT R: CTTGCATTCACTTCCGGTGT	NM_204153.2	197
IL4	F: TTCCTGCGTCAAGATGAACG R: CGCATGTTGAGGAAGAGACC	NM_001007079.2	144
IL6	F: GGCTTCGACGAGGAGAAATG R: AGAGACTCGACGTTCTGCTT	NM_204628.2	116
IL10	F: GAGATGCTGCGCTTCTACAC R: TCTGCTTGATGGCTTTGCTC	NM_001004414.4	172
IL-1β	F: GCATCAAGGGCTACAAGCTC R: GTCCAGGCGGTAGAAGATGA	NM_204524.2	134
TNF-α	F: GGACAGCCTATGCCAACAAG R: CGCTCCTGACTCATAGCAGA	NM_204267.2	150
INF-γ	F: GTGGAGCTTTGACGAGCACT R: ATTCCCAGCATACGACAGGGT	NM_010511.3	105

^1^ IL-2=interleukin-2; IL-4=interleukin-4; IL-6=interleukin-6; IL-10=interleukin-10; IL-1β=interleukin-1β; TNF-α = tumor necrosis factor alpha; IFN-γ = interferon-γ.

### 16 S rRNA gene sequencing analysis

Total genomic DNA from 0.5 g of each cecal sample was extracted using the OMEGA Soil DNA Kit (M5636-02, Omega Bio-Tek, Norcross, GA, USA) according to the manufacturer’s instructions and stored at −20 °C until analysis. The concentration and quality of the extracted DNA were assessed using a NanoDrop NC2000 spectrophotometer (Thermo Fisher Scientific, Waltham, MA, USA) and agarose gel electrophoresis, respectively.

The V3–V4 hypervariable region of the bacterial 16S rRNA gene was amplified by PCR using the forward primer 338F (5′-ACTCCTACGGGAGGCAGCAG-3′) and the reverse primer 806R (5′-GGACTACHVGGGTWTCTAAT-3′). Sample-specific 7-bp barcodes were incorporated into the primers for multiplex sequencing. Each 25 μL PCR reaction mixture contained 5 μL of 5 × buffer, 0.25 μL of Fast Pfu DNA Polymerase (5 U/μL), 2 μL of dNTPs (2.5 mM each), 1 μL of each forward and reverse primer (10 μM), 1 μL of DNA template, and 14.75 μL of sterile ddH₂O. The thermal cycling conditions were as follows: initial denaturation at 98 °C for 5 min; 25 cycles of denaturation at 98 °C for 30 s, annealing at 53 °C for 30 s, and extension at 72 °C for 45 s; followed by a final extension at 72 °C for 5 min. The resulting PCR amplicons were purified using Vazyme VAHTSTM DNA Clean Beads (Vazyme, Nanjing, China) and quantified with the Quant-iT PicoGreen dsDNA Assay Kit (Invitrogen, Carlsbad, CA, USA). After individual quantification, amplicons were pooled in equimolar amounts. Paired-end sequencing (2 × 250 bp) was performed on an Illumina NovaSeq 6000 platform using the NovaSeq 6000 SP Reagent Kit (500 cycles) at Shanghai Personal Biotechnology Co., Ltd. (Shanghai, China).

### Bioinformatics and statistical analysis

Raw paired-end reads were demultiplexed based on their unique barcodes, followed by the removal of barcode and primer sequences. Bioinformatics processing, including sequence quality filtering, merging, and amplicon sequence variant (ASV) clustering, was conducted primarily using the QIIME2 pipeline and supported by specific R packages (v3.2.0). Part of this process, specifically sequence splicing, quality control, and operational taxonomic unit (OTU) clustering, was performed on the online Genes Cloud platform (https://www.genescloud.cn/; Shanghai Personal Biotechnology Co. Ltd., China).

Alpha diversity indices, including the Chao1 richness estimator, observed species, Shannon index, and Simpson index, were calculated from the ASV table in QIIME2. Ranked abundance curves were plotted to visualize and compare the richness and evenness of ASVs across samples. Beta diversity was analyzed to examine structural differences in microbial communities using principal coordinate analysis (PCoA) based on unweighted UniFrac distances. To identify differentially abundant taxa across groups at the phylum and genus levels, linear discriminant analysis effect size (LEfSe) was performed with default parameters, applying a linear discriminant analysis (LDA) score threshold of > 3.5 to estimate the effect size of each significant feature.

ASV-level alpha-diversity indices, such as Chao1 richness estimator, observed species, Shannon diversity index, and Simpson index were calculated using the ASV table in QIIME2. ASV-level ranked abundance curves were generated to compare the richness and evenness of ASVs among samples. Beta-diversity analysis was assessed to investigate the structural variation of microbial communities via principal coordinate analysis (PCoA) based on unweighted (assessment of community structure by considering the abundance of OTU). LEfSe (Linear discriminant analysis effect size) was performed to detect differentially abundant taxa at phylum and genus level across groups using the default parameters. Linear discriminant analysis (LDA) >3.5 was used as the criterion to estimate the effect size of each differentially abundant OTU.

### Data analysis

All experimental data were analyzed using GraphPad Prism version 9.5, with the replicate pen serving as the experimental unit. Prior to analysis, data normality and homogeneity of variances were verified using appropriate tests. For normally distributed data with homogeneous variances, one-way analysis of variance (ANOVA) was performed. If a significant overall effect (*P* < 0.05) was detected, differences among group means were further compared using Duncan's multiple range test. Data are presented as the mean ± standard error of the mean (SEM). A single asterisk (*) denotes a statistically significant difference at *P* < 0.05, and a double asterisk (**) denotes *P* < 0.01.

## Results

### Growth performance

We analyzed the growth performance of broilers during the starter phase (1-21 days), finisher phase (22-42 days), and the entire growth period (1-42 days).

**Fig. 1:**
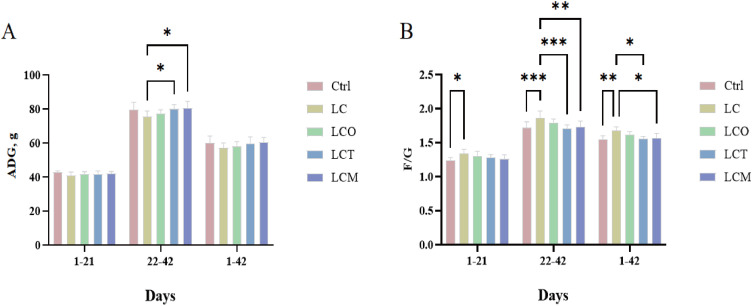
Effects of OEO, OEC and OEL on the growth performance of broilers (*n* = 6). (A) average daily gain (ADG); (B) overall feed conversion ratio (F/G).

Growth performance analysis revealed that the low-energy diet itself increased the F/G compared to the normal-energy control (Ctrl) (Fig. 1B). Notably, this adverse effect was effectively mitigated by specific additives: both the oregano-clove composite (OEC; LCT group) and the oregano-lauric acid composite (OEL; LCM group) significantly reduced the F/G (*P* < 0.05), restoring it to a level statistically similar to the Ctrl group. Regarding growth, supplementation with OEL (LCM group) uniquely improved the average daily gain (ADG) during the finisher phase (d 22-42) compared to the low-energy control (LC) (*P* < 0.05; Fig. 1A), representing a 1.02% numerical increase over the Ctrl group. No significant effects of additive supplementation on ADG were observed during the starter phase or over the entire trial period.

### Antioxidative capacity of jejunal and ileal mucosa

**Fig. 2:**
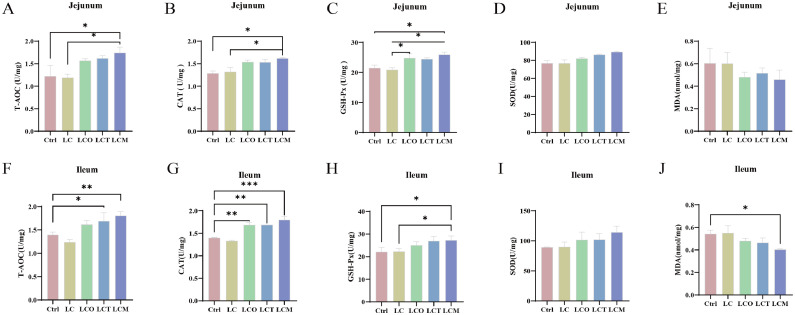
Antioxidant levels in the jejunum (A-E) and ileum (F-J) of broilers in response to phytogenic essential oils. (A) the T-AOC level in the jejunum; (B) the CAT level in the jejunum; (C) the GSH-Px level in the jejunum; (D) the SOD level in the jejunum; (E) the MDA level in the jejunum; (F) the T-AOC level in the ileum; (G) the CAT level in the ileum; (H) the GSH-Px level in the ileum; (I) the SOD level in the ileum; (J) the MDA level in the ileum.

Reducing the dietary energy level (LC group) did not significantly affect the antioxidative capacity in the jejunal or ileal mucosa compared to the normal-energy control (Ctrl) (*P* > 0.05; Fig. 2).

In the jejunum (Fig. 2A-E), supplementation with the oregano-clove composite significantly enhanced the activities of T-AOC, CAT, and GSH-Px compared to both the Ctrl and LC groups. Oregano essential oil also increased GSH-Px activity under low-energy conditions (*P* < 0.05). SOD activity and MDA content remained unchanged across all groups.

In the ileum (Fig. 2F-J), adding OEC or OEL to the low-energy diet significantly improved T-AOC activity compared to the Ctrl group. All three essential oil supplements (OEO, OEC, and OEL) significantly enhanced CAT activity. Furthermore, essential oil supplementation markedly reduced ileal MDA content (*P* < 0.05), with the lowest value observed in the LCT (OEC) group. No significant changes were observed in ileal GSH-Px or SOD activity among groups.

### Mucosal immunity in the jejunum and ileum

**Fig. 3:**
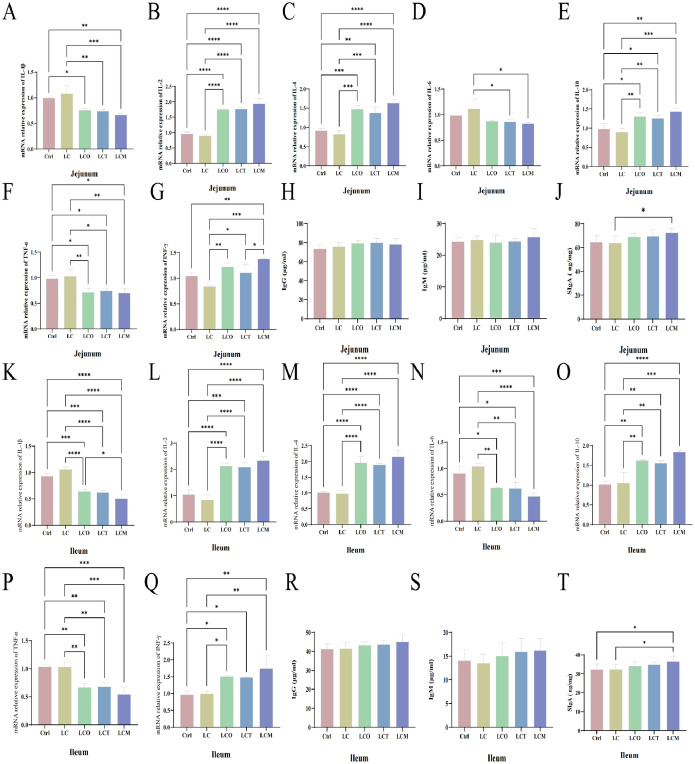
Phytogenic essential oils modulate intestinal immunity in broilers, including mRNA expression of immune factors and antibodies in the jejunum (A-J) and ileum (K-T). (A) the IL-1β level in the jejunum; (B) the IL-2 level in the jejunum; (C) the IL-4 level in the jejunum; (D) the IL-6 level in the jejunum; (E) the IL-10 level in the jejunum; (F) the TNF-α level in the jejunum; (G) the INF-γ level in the jejunum; (H)the IgG level in the jejunum; (I) the IgM level in the jejunum; (J) the SIgA level in the jejunum; (K) the IL-1β level in the ileum; (L) the IL-2 level in the ileum; (M) the IL-4 level in the ileum; (N) the IL-6 level in the ileum; (O) the IL-10 level in the ileum; (P) the TNF-α level in the ileum; (Q) the INF-γ level in the ileum; (R)the IgG level in the ileum; (S) the IgM level in the ileum; (T) the SIgA level in the ileum.

Fig. 3 displays the expression results of immune-related genes and cytokines in the jejunum and ileum. Compared to the Ctrl and LC groups, dietary supplementation with phytogenic essential oils significantly up-regulated the expression of IL-2, IL-4, and IL-10 in both the jejunum and ileum, as well as the expression of IFN-γ in the ileum (Fig. 3B, C, E, L, M, O, Q). Concurrently, they significantly down-regulated the expression of TNF-α in both the jejunum and ileum, and the expression of IL-1β, IL-6, and IFN-γ in the ileum (*P* < 0.05) (Fig. 3F, K, N, P). Compared to the Ctrl group, the addition of OEO and OEL significantly reduced the level of IL-1β in the jejunum. Similarly, compared to the LC group, supplementation with OEC and OEL significantly decreased the level of IL-1β in the jejunum (Fig. 3A). All phytogenic essential oil groups significantly up-regulated the level of IFN-γ and down-regulated the level of IL-6 in the jejunum (*P* < 0.05) (Fig. 3D, G).

Notably, neither reducing dietary energy levels nor supplementing with phytogenic essential oils had a significant effect on IgG and IgM levels (Fig. 3H, I, R, S). However, under low-energy conditions, OEL supplementation enhanced the expression of SIgA in both the jejunum and ileum (Fig. 3J, T).

### Intestinal morphology and histomorphology

**Fig. 4:**
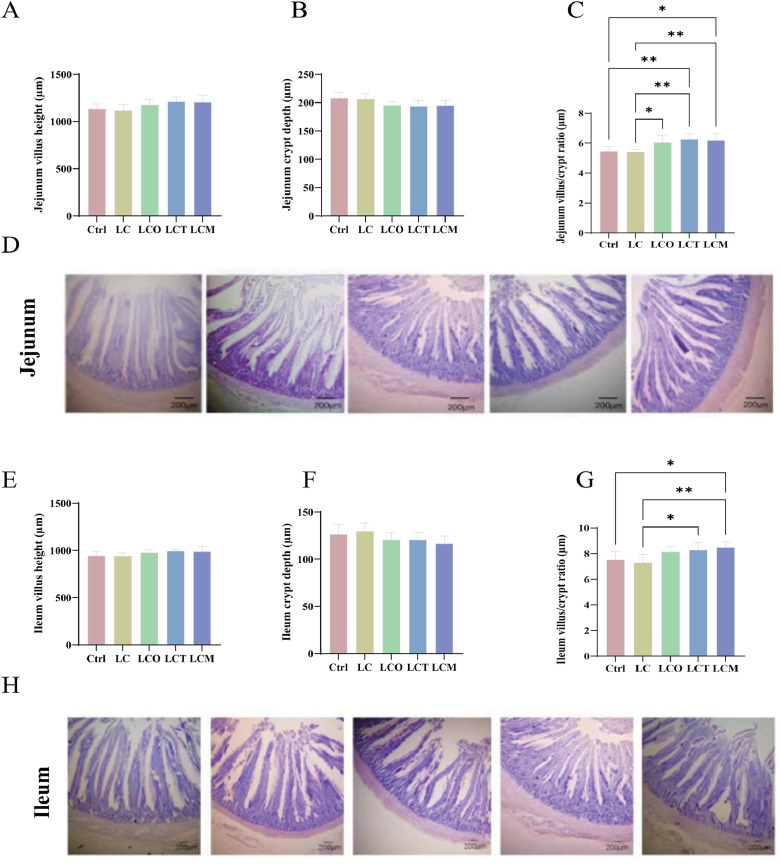
Effects of phytogenic essential oils on histomorphological parameters of jejunum and ileum in broilers. Histomorphometric analysis of jejunum (A-D) and ileum (E-H) by Hematoxylin & Eosin (H&E) staining. Scale bar: 200 μm. (A) Jejunum villus height; (B) Jejunum crypt depth; (C) Jejunum villus/ crypt radio; (D) Jejunal histomorphology; (E) Ileum villus height; (F) Ileum crypt depth; (G) Ileum villus/ crypt radio; (H) Ileal histomorphology.

Histological analysis of intestinal morphology is presented in Fig. 4. Compared to both the Ctrl and LC groups, dietary supplementation with phytogenic essential oils tended to increase jejunal villus height and decrease crypt depth (Fig. 4A, B). Consequently, the villus height-to-crypt depth ratio (VH:CD) was significantly elevated in the supplemented groups (*P* < 0.05; Fig. 4C), except for the OEO group, in which the increase relative to the Ctrl group did not reach statistical significance.

A similar improvement in ileal morphology was observed: all three additives significantly increased the VH:CD ratio compared to the LC group (Fig. 4E, F). Representative micrographs (Fig. 4D, H) revealed that the intestinal villi in the essential oil-supplemented groups were structurally intact, tightly arranged, and showed no evident epithelial exfoliation or inflammatory cell infiltration.

### Fecal microbiota

**Fig. 5:**
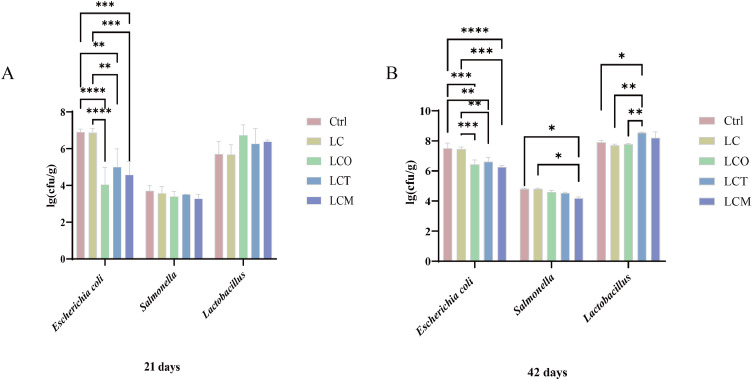
Effect of phytogenic essential oils on the fecal microbiota of broilers at 21 and 42 days of age.

The results of fecal bacterial enumeration are presented in Fig. 5. Compared to both the normal-energy control (Ctrl) and low-energy control (LC) groups, dietary supplementation with oregano essential oil (OEO), oregano-clove composite (OEC), or oregano-lauric acid composite (OEL) significantly reduced the fecal counts of *Escherichia coli* on both days 21 and 42 (*P* < 0.05).

Regarding *Salmonella*, supplementation showed a decreasing trend on day 21. By day 42, a significant reduction was observed specifically in the OEL-supplemented (LCM) group compared to both the Ctrl and LC groups (*P* < 0.05). For *Lactobacillus*, an increasing trend was noted on day 21, and by day 42, the OEC-supplemented (LCT) group exhibited a significant increase in counts compared to the control groups (*P* < 0.05).

### Diversity of cecal microbiota

**Fig. 6:**
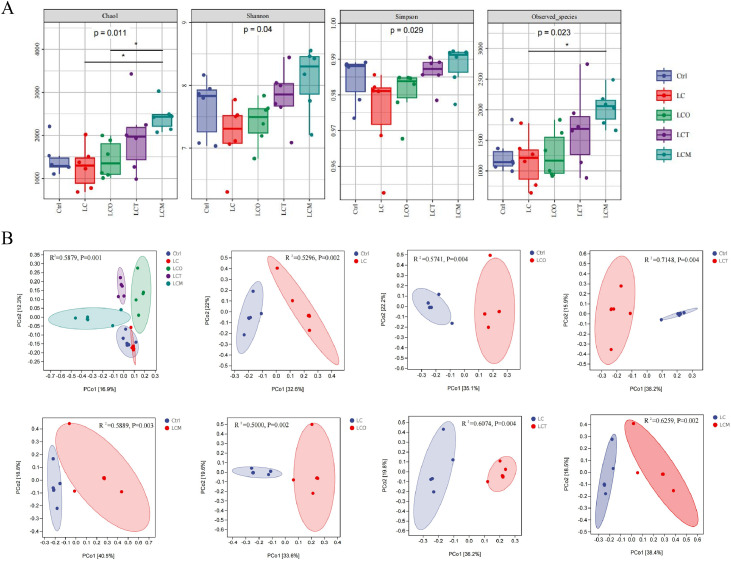
Effect of phytogenic essential oils on the cecal microbial community and structure of cecum by Alpha (α) and Beta (β) diversity analysis of from broilers. (A) Chao 1, Shannon, Simpson, Observed species index of ASV level. (B) PCoA plots assessed by Adonis analysis among these treatments.

16S rRNA gene sequencing targeting the V3–V4 region was performed on cecal samples from all groups (Ctrl, LC, LCO, LCT, LCM; *n* = 6 per group). Alpha diversity analysis (Fig. 6A) showed that the Chao1 and Observed species indices were significantly higher in the LCM group (supplemented with OEL) than in the LC and LCO groups (*P* < 0.05). Supplementation with OEL also significantly increased the Observed species index compared to the LC group. No significant differences were detected in the Shannon or Simpson indices among groups.

Beta diversity was assessed by principal coordinate analysis (PCoA) based on Bray-Curtis distances (Fig. 6B). The PCoA plot revealed distinct clustering of microbial communities according to dietary treatment. This was statistically supported by PERMANOVA (Adonis), which confirmed that dietary treatment (including both the low-energy diet and essential oil supplementation) explained a significant portion of the variance in community structure (*P* < 0.05). Pairwise comparisons indicated significant differences in β-diversity between the Ctrl group and all other groups (LC, LCO, LCT, LCM), as well as between the LC group and all essential oil-supplemented groups (LCO, LCT, LCM).

### Composition of cecal microbiota

**Fig. 7:**
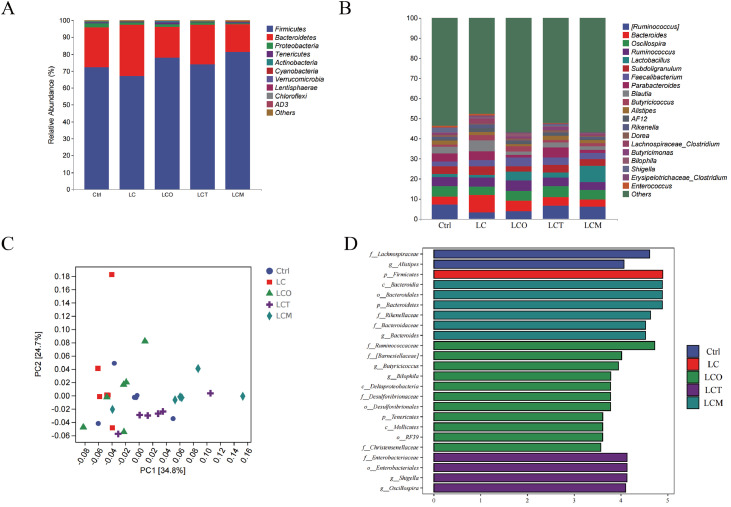
Effects of the dietary supplementation of phytogenic essential oils on the microbiological analysis of the broilers. The microbial community structure: (A) the phylum level; (B) the genus level; (C) Principal component analysis (PCA) of the dissimilarity among the microbial samples at the genus levels. The values of axes 1 and 2 are the percentages that can be explained by the corresponding axis; (D) Linear discriminant analysis effect size (LEfSe) was performed to determine the difference in abundance among these treatments, the LDA value distribution histogram shows bacterial community or species (Biomarker) with LDA score greater than 3.5.

The results of the relative abundance analysis are shown in Fig. 7. At the phylum level (Fig. 7A), the cecal microbiota was predominantly composed of Firmicutes and Bacteroidota, which together accounted for over 60% of the total community. Compared to the Ctrl group (72.12%), the low-energy diet (LC group) reduced the relative abundance of Firmicutes to 67.09%. Supplementation with phytogenic essential oils based on this low-energy diet restored and increased the abundance of Firmicutes, with the most pronounced effect observed in the LCM (OEL) group (81.15%). At the genus level (Fig. 7B), the predominant taxa included *Ruminococcus, Bacteroides, Oscillospira*, and *Lactobacillus*. Supplementation with OEO, OEC, or OEL significantly increased the relative abundance of *Lactobacillus* compared to both the Ctrl and LC groups.

Principal component analysis (PCA) was performed to visualize overall community differences (Fig. 7C). The Ctrl, LC, and LCO groups clustered closely, while the LCT (OEC) and LCM (OEL) groups formed distinct clusters, clearly separating from the others. Specific bacterial taxa associated with the treatments were identified by linear discriminant analysis effect size (LEfSe) (LDA score > 3.5, *P* < 0.05). A total of 24 significant biomarkers were identified at the genus level (Fig. 7D). The major discriminative taxa in the Ctrl group included *f_Lachnospiraceae* and *g_Alistipes*, whereas the LC group was characterized by enrichment of *p_Firmicutes*. Differential enrichment patterns were observed in the supplemented groups: the OEO group enriched *c_Bacteroidia, o_Bacteroidales*, and *p_Bacteroidetes*; the OEC group enriched *f_Ruminococcaceae, g_Barnesiellaceae*, and *g_Bilophila*; and the OEL group enriched *f_Enterobacteriales, g_Shigella*, and *g_Oscillospira*.

## Discussion

Metabolizable energy (ME) is a critical component in broiler nutrition, serving as the primary energy source for both maintenance and productive functions such as growth (Gopinger et al., 2017). Dietary ME is prioritized for meeting maintenance requirements before being allocated to growth. Consequently, a reduction in dietary ME levels typically compromises growth performance, often manifesting as an elevated F/G. Consistent with this, our study observed higher ADG and lower F/G in broilers fed the basal diet compared to those on a reduced-energy diet from day 1 to 42. This may explain why the reduction in ME density (by 210 kJ/kg) resulted in increased F/G across different growth phases in the present study.

Oregano essential oil (OEO) exhibits antibacterial and anti-inflammatory properties due to its active components, thymol and carvacrol, while also improving nutrient utilization and gut health (Falleh et al., 2020; Pirgozliev et al., 2019). The study found that both OEC and OEL improved broiler feed conversion efficiency, consistent with previous research, potentially due to synergistic effects among the three components on growth performance and intestinal health (Christaki et al., 2020; Youssefi et al., 2023; Zaazaa et al., 2022). Notably, no significant differences in feed intake were observed among the groups, which may be attributed to broilers' weaker taste perception resulting from the absence of the sweet taste receptor gene T1R2 (Shi and Zhang, 2006). Oregano essential oil and lauric acid enhance nutrient utilization through multiple mechanisms: lauric acid promotes growth and immune function by regulating lipid metabolism and gut microbiota (Wu et al., 2021b), while clove essential oil improves intestinal amylase and lipase activity, modulates microbial composition, inhibits specific pathogens, reduces immune expenditure, and directs more nutrients toward meat production (Seidavi et al., 2021). Research indicates that compound essential oils derived from oregano, thyme, and other herbs can improve production performance through a dual mechanism involving local intestinal effects and systemic metabolic regulation (Hu et al., 2023). The advantage of essential oil preparations lies in their multifaceted biological activities, including intestinal regulation, antioxidant, and immunomodulatory effects (Reyer et al., 2017). This study also found that essential oil supplementation enhanced antioxidant capacity, anti-inflammatory activity, improved intestinal morphology, and modulated microbial composition, collectively promoting nutrient digestion and absorption.

In broiler health management, maintaining the balance between oxidation and antioxidation in the body is crucial. Previous studies have shown that plant-derived essential oils can effectively inhibit endogenous oxidation by activating the antioxidant defense system (Tian and Piao, 2019). Supplementation with oregano essential oil (Li et al., 2024; Othman et al., 2022), as well as a composite essential oil containing monoglyceride laurate and cinnamaldehyde (Zheng et al., 2023), has been demonstrated to enhance the systemic antioxidant capacity in poultry. Additionally, phenolic compounds in clove essential oil significantly inhibit hydroxyl radicals (Jirovetz et al., 2006). This study further validates the above findings: Under conditions of basal diet and reduced metabolizable energy (ME) density, the addition of OEO (oregano essential oil), OEC (oregano with clove essential oils), and OEL (oregano essential oil with lauric acid) increased the activities of T-AOC, CAT, GSH-Px, and SOD in broilers. The main active components of these essential oils, such as thymol, carvacrol, eugenol, and lauric acid, are key to their antioxidant effects (Li et al., 2022). Consistent with this, Liu et al. (Liu et al., 2022) reported that a compound essential oil containing similar components not only increased serum SOD and GPX activities and reduced MDA content but also enhanced GPX activity and the expression of its regulatory gene Nrf2 in the jejunum. OEL has also been reported to improve antioxidant stability (Anuar et al., 2023). Most importantly, this study observed a synchronous decrease in MDA levels alongside an increase in the activities of multiple antioxidant enzymes, indicating that oregano essential oil and its compounds exert a synergistic antioxidant effect in broilers, confirming the rationality and effectiveness of their formulation ratio.

Gut health is fundamental to the production performance of broilers, with the intestinal epithelial barrier playing a dual critical role in nutrient absorption and immune defense (Chen et al., 2018). Zhou et al. (Zhou et al., 2023) reported that low metabolizable energy (ME) diets (2850 vs. 3050 kcal/kg) significantly reduced serum IgM levels in broilers, indicating that energy levels influence immune responses. This study also found that although ME restriction did not lead to a significant decrease in jejunal and ileal mucosal immunoglobulins, its effects were modulated by dietary composition and the physiological state of the animals. Under normal rearing conditions, inflammatory responses in broilers were not highly activated, providing potential regulatory room for essential oil interventions. Ruan et al. (Ruan et al., 2021) demonstrated that oregano essential oil (OEO) increased ileal secretory IgA (SIgA) and reduced TNF-α levels, while Feng et al. (Feng et al., 2021) further confirmed that OEO downregulated the gene expression of TLR-4 and pro-inflammatory cytokines. Lauric acid (LA) exhibited anti-inflammatory potential by bidirectionally regulating immunoglobulins and inflammatory factors (Wang et al., 2022). In addition, clove essential oil has also been shown to improve the overall immune status of broilers (Islam and Sultana, 2023). This study revealed that supplementation with OEO, OEL, and OEC significantly increased intestinal SIgA levels and differentially modulated cytokine expression: while downregulating pro-inflammatory factors such as IL-6, IL-1β, and TNF-α, they upregulated anti-inflammatory factors including IL-2, IL-4, and IL-10 (Wu et al., 2021a; Yeom et al., 2022). These findings indicated that plant essential oils not only alleviate inflammatory responses but may also indirectly enhance the body's antioxidant defense by modulating immune–antioxidant cross-talk pathways.

The intestinal epithelial structure is crucial for nutrient digestion and absorption, and its morphological changes serve as important indicators of intestinal health (Greig and Cowles, 2017). Studies have shown that dietary supplementation with oregano essential oil, lauric acid, and clove bud powder can improve intestinal morphology in broilers, specifically by increasing villus height, reducing crypt depth, and thereby enhancing the villus height-to-crypt depth ratio (VH:CD) (Johnson et al., 2025). In In the experiment, the addition of OEO, OEC, and OEL effectively improved the VH:CD in the jejunum and ileum. This improvement may be attributed to the synergistic effects of active components such as carvacrol (Brenes and Roura, 2010), thymol (Li et al., 2024), eugenol (Boa Ventura et al., 2025), and lauric acid (Jadhav et al., 2021), which collectively exert antimicrobial, anti-inflammatory, and antioxidant activities. Although previous studies reported no significant effect of clove extract on intestinal morphology (Chakma et al., 2020)—possibly due to differences in extraction methods, plant parts used, or rearing conditions—OEC in this study still demonstrated a certain degree of improvement, suggesting functional synergism among its components. As a medium-chain fatty acid, lauric acid can be directly utilized by intestinal epithelial cells as an energy source, promoting intestinal development and structural integrity (Jadhav et al., 2021). The morphological improvements in the intestinal epithelium help defend against pathogen invasion, enhance digestive enzyme secretion, and improve nutrient utilization efficiency (Stefanello et al., 2019), ultimately leading to enhanced growth performance in broilers.

The functional status of intestinal health is regulated by a combination of disease, environment, and dietary composition, with the gastrointestinal microbiota playing a central role (Shang et al., 2018). Gut microorganisms are involved not only in nutrient metabolism but also in regulating the development of the host's digestive and immune systems (Pan and Yu, 2014). The symbiotic relationship between the host and the microbiota forms an important physiological basis for broiler health and production performance. Studies have shown that the addition of blended essential oils to feed can effectively optimize the cecal microbiota structure in broilers, specifically by reducing coliform counts and increasing the proportions of *Enterococcus* and *Lactobacilli* (Ruan et al., 2021). Among these, *Enterococcus faecium* specifically antagonizes *Salmonella*, while *Lactobacilli* effectively inhibit the colonization of pathogens such as *Salmonella, Clostridium perfringens*, and pathogenic *E. coli* (Mead, 2000). Other studies have reported that dietary supplementation with essential oils and organic acids significantly reduces *C. perfringens* counts but has no significant effect on *E. coli* or *Lactobacilli* levels (Si et al., 2009). Similarly, although clove essential oil increases *Lactobacilli* counts, it does not significantly alter *Salmonella* or *E. coli* levels (Chakma et al., 2020). This study further confirms that supplementation with OEC and OEL significantly reduces the abundance of *E. coli* and *Salmonella* in the cecum while increasing the proportion of *Lactobacilli*, indicating their positive role in modulating the gut microbiota and promoting broiler health and growth.

To further investigate the regulatory mechanisms of oregano essential oil and its compounds (OEO, OEC, OEL) on the gut microbiota (Michaudel and Sokol, 2020), this study analyzed their effects on the composition of the cecal microbiota. The results showed that all three additives significantly increased the richness and diversity of the gut microbial community, which is consistent with previous findings (Hernández-Patlán et al., 2019; Kim et al., 2023) that OEO improves intestinal barrier integrity and modulates microbiota composition. Based on PCoA and LEfSe analyses, at the phylum level, OEO and OEL treatments increased the relative abundance of *Firmicutes* and decreased the proportion of *Bacteroidetes* (Ballou et al., 2016), thereby raising the *Firmicutes*/*Bacteroidetes* ratio, which contrasted with the trend observed in the energy-restricted group. At the genus level, OEL treatment significantly increased the abundance of *Ruminococcus* and *Lactobacillus* (Ding et al., 2017), while reducing the proportion of *Bacteroides. Lactobacillus* helps maintain intestinal immune homeostasis, and *Ruminococcus* produces short-chain fatty acids through the degradation of dietary fiber, providing energy for the host and enhancing intestinal barrier integrity (Karlsson et al., 2011; Zakharzhevskaya et al., 2017). The optimization of this microbial structure was consistent with higher levels of antioxidant and anti-inflammatory activity, as well as improved growth performance (Zhu et al., 2021).

This study primarily reveals the pathway through which essential oils indirectly improve feed efficiency by enhancing intestinal health. To systematically elucidate the complete energy-saving mechanism, future work needs to bridge intestinal health indicators with direct evidence of energy metabolism, focusing on measuring apparent metabolizable energy, nutrient digestibility, key metabolic hormone levels, and quantifying energy loss through indirect calorimetry. Additionally, more comprehensive independent analytical determinations of dietary nutritional levels, including gross energy and crude fat, are required. By integrating these indicators, it may be possible to construct a complete physiological pathway from intestinal function regulation to efficient energy utilization in the organism, thereby directly verifying the energy-saving effects of essential oils at both digestive and metabolic levels.

## Conclusion

This study evaluated the application of oregano essential oil (OEO) and its compound in broilers fed low‑metabolizable‑energy diets. We focused on their effects on growth performance and intestinal health. Specifically, oregano essential oil and its composites modulated the gut microbiota structure, enhanced intestinal antioxidant capacity, optimized mucosal immune status, and improved intestinal morphology. These effects mitigated the adverse impacts of energy reduction and increased feed conversion efficiency. The findings reveal the promising potential of the oregano essential oil‑lauric acid composite in improving production performance and intestinal health of broilers under low‑energy diets, offering a novel strategy for developing green and energy‑saving feed additives.

**Availability of data and materials**: The original contributions presented in the study are included in the article, further inquiries can be directed to the corresponding author.

## Funding

This study was partially supported by the National Key R & D program of China (2017YFE0135200) which was a joint-research program between China and Greece and the first batch of scientific and technological research projects of 10.13039/501100012131Liaoning Provincial Science and Technology Department (2021JH1/10400033) and Liaoning Provincial Science and Technology Program Project (2025JH5/10400047).

## Consent for publication

Not applicable.

## CRediT authorship contribution statement

**Xiaotong Li:** Writing – review & editing, Writing – original draft, Visualization, Validation, Supervision, Software, Project administration, Methodology, Investigation, Formal analysis, Data curation. **Sihuan Wang:** Writing – review & editing, Visualization, Validation, Supervision, Software, Project administration, Methodology, Investigation, Formal analysis, Data curation. **Libo Zhang:** Writing – review & editing, Validation, Methodology, Data curation. **Desheng Li:** Writing – review & editing, Validation, Methodology, Data curation. **Huiying Li:** Writing – review & editing, Validation, Methodology, Data curation. **Qiyue Zhang:** Writing – review & editing, Validation, Methodology, Data curation. **Changmin Jin:** Writing – review & editing, Validation, Methodology, Data curation. **Jiali Wang:** Writing – review & editing, Validation, Investigation. **Jun Wang:** Writing – review & editing, Validation, Investigation. **Jie Han:** Writing – review & editing. **Fengxia Liu:** Writing – review & editing. **Shunshun Jin:** Writing – review & editing, Validation. **Lizhi Jin:** Writing – review & editing. **Donghui Shi:** Writing – review & editing, Supervision, Software, Methodology, Investigation.

## Disclosures

The authors declare that the research was conducted in the absence of any commercial or financial relationships that could be construed as a potential conflict of interest.

## References

[bib0001] Afrendi E., Prastya M.E., Astuti R.I., Wahyuni W.T., Batubara I. (2023). Bioactivity of the ethanol extract of clove (Syzygium aromaticum) as antitoxin. Int. J. Food Sci..

[bib0002] Anuar N.S., Shafie S..A., Maznan M.A.F., Zin N., Azmi N.A.S., Raoof R.A., Myrzakozha D., Samsulrizal N. (2023). Lauric acid improves hormonal profiles, antioxidant properties, sperm quality and histomorphometric changes in testis and epididymis of streptozotocin-induced diabetic infertility rats. Toxicol. Appl. Pharmacol..

[bib0003] Ballou A.L., Ali R..A., Mendoza M.A., Ellis J.C., Hassan H.M., Croom W.J., Koci M.D. (2016). Development of the chick microbiome: how early exposure influences future microbial diversity. Front. Vet. Sci..

[bib0004] Boa Ventura P.d.V., Chaves A.C., Pereira M.S., de Carvalho F.J.N., da Silva B.F., Costa R.A., d. C. Teixeira R.S., Maciel W.C., Carneiro V.A. (2025). Exploring plant compounds as enhancers of ciprofloxacin activity against planktonic and biofilm cells of poultry-related enterobacteriaceae. Microb. Pathog..

[bib0005] Brenes A., Roura E. (2010). Essential oils in poultry nutrition: main effects and modes of action. Anim. Feed. Sci. Technol..

[bib0007] Chakma, J., A. K. Samanta, T. K. Dutta, and R. S. J. I. J. o. A. H. Arya. 2020. Effect of supplementing Moringa oleifera leaf extract and clove bud oil to the diet on microflora population and intestinal morphology of broiler birds.

[bib0008] Chen J.L., Yu B.., Chen D.W., Huang Z.Q., Mao X.B., Zheng P., Yu J., Luo J.Q., He J. (2018). Chlorogenic acid improves intestinal barrier functions by suppressing mucosa inflammation and improving antioxidant capacity in weaned pigs. J. Nutr. Biochem..

[bib0006] Çenesiz A.A., Çiftci İ. (2020). Modulatory effects of medium chain fatty acids in poultry nutrition and health. World's. Poult. Sci. J..

[bib0009] Cheng C., Liu Z., Zhou Y., Wei H., Zhang X., Xia M., Deng Z., Zou Y., Jiang S., Peng J. (2017). Effect of oregano essential oil supplementation to a reduced-protein, amino acid-supplemented diet on meat quality, fatty acid composition, and oxidative stability of Longissimus thoracis muscle in growing-finishing pigs. Meat. Sci..

[bib0010] Christaki E., Giannenas I., Bonos E., Florou-Paneri P., Florou-Paneri P., Christaki E., Giannenas I. (2020). Chapter 2 - Innovative Uses of Aromatic Plants as Natural Supplements in Nutrition.

[bib0011] Ding Y.H., Qian L..Y., Pang J., Lin J.Y., Xu Q., Wang L.H., Huang D.S., Zou H. (2017). The regulation of immune cells by Lactobacilli: a potential therapeutic target for anti-atherosclerosis therapy. Oncotarget..

[bib0012] Falleh H., Ben Jemaa M., Saada M., Ksouri R. (2020). Essential oils: a promising eco-friendly food preservative. Food Chem..

[bib0013] Fauzya A.F., Astuti R..I., Mubarik N.R. (2019). Effect of ethanol-derived clove leaf extract on the oxidative stress response in yeast schizosaccharomyces pombe. Int. J. Microbiol..

[bib0014] Felici M., Tugnoli B., Ghiselli F., Baldo D., Ratti C., Piva A., Grilli E. (2023). Investigating the effects of essential oils and pure botanical compounds against Eimeria tenella in vitro. Poult. Sci..

[bib0015] Feng J., Lu M.Y., Wang J., Zhang H.J., Qiu K., Qi G.H., Wu S.G. (2021). Dietary oregano essential oil supplementation improves intestinal functions and alters gut microbiota in late-phase laying hens. J. Anim. Sci. Biotechnol..

[bib0016] Giannenas I., Bonos E., Skoufos I., Tzora A., Stylianaki I., Lazari D., Tsinas A., Christaki E., Florou-Paneri P. (2018). Effect of herbal feed additives on performance parameters, intestinal microbiota, intestinal morphology and meat lipid oxidation of broiler chickens. Br. Poult. Sci..

[bib0017] Gopinger E., Krabbe E.L., Surek D., Lopes L.S., Avila V.S. (2017). Live performance, carcass, and bone quality responses of grower and finisher broilers to dietary metabolizable energy levels. Braz. J. Poult. Sci..

[bib0018] Greig C.J., Cowles R.A. (2017). Muscarinic acetylcholine receptors participate in small intestinal mucosal homeostasis. J. Pediatr. Surg..

[bib0019] Hernández-Patlán D., Solís-Cruz B., Pontin K.P., Hernández-Velasco X., Merino-Guzmán R., Adhikari B., López-Arellano R., Kwon Y.M., Hargis B., Arreguin-Nava M.A., Téllez-Isaías G., Latorre J.D.J.F.i.V.S. (2019).

[bib0021] Hu Q., Zhou M., wei S. (2018). Progress on the antimicrobial activity research of clove oil and eugenol in the food antisepsis field. J. Food Sci..

[bib0020] Hu J., Zhu H., Feng Y., Yu M., Xu Y., Zhao Y., Zheng B., Lin J., Miao W., Zhou R., Cullen P.J. (2023). Emulsions containing composite (clove, oregano, and cinnamon) essential oils: phase inversion preparation, physicochemical properties and antibacterial mechanism. Food Chem..

[bib0022] Inci H., Sogut B., Gokdogan O., Ayasan T., Sengul T. (2016). Determining the energy usage efficiency and economic analysis of broiler chickens raised under organic conditions. Indian J. Anim. Sci..

[bib0023] Islam R., Sultana N. (2023). Efficacy of clove and tulsi supplementation in drinking water in broiler immunity. Vet. Med. Sci..

[bib0024] Jadhav P., Manwar S., Khose K., Wade M., Gole M., Langote G. (2021). Effect of medium chain fatty acids as replacement to antibiotics in diets on growth performance and gut health in broiler chicken. Indian J. Anim. Res..

[bib0025] Jirovetz L., Buchbauer G., Stoilova I., Stoyanova A., Krastanov A., Schmidt E. (2006). Chemical composition and antioxidant properties of clove leaf essential oil. J. Agric. Food Chem..

[bib0026] Johnson A.M., Anderson M..G., Arguelles-Ramos M., Ali A.B.A. (2025). The effects of dietary oregano essential oil on production, blood parameters, and egg quality of laying hens during the early lay phase. Anim. - Open Space.

[bib0027] Karlsson F.H., Ussery D..W., Nielsen J., Nookaew I. (2011). A closer look at bacteroides: phylogenetic relationship and genomic implications of a life in the human gut. Microb. Ecol..

[bib0028] Kim H.J., Kim H..S., Yun Y.S., Kang H.K. (2023). Effect of Bacillus subtilis and oregano oil on performance, gut microbiome, and intestinal morphology in pullets. Anim.: An Open Access J. From MDPI.

[bib0029] Lara L.J., Rostagno M.H. (2013). Impact of heat stress on poultry production. Anim.: An Open Access J. From MDPI.

[bib0031] Li Y.X., Erhunmwunsee F.., Liu M., Yang K.L., Zheng W.F., Tian J. (2022). Antimicrobial mechanisms of spice essential oils and application in food industry. Food Chem..

[bib0030] Li Y., Li C., Zhang Y., Everaert N., Comer L., Huang L., Jiao N., Yuan X., Yang W., Jiang S. (2024). Effects of dietary supplementation with cocrystals of thymol and carvacrol on quality, nutrient composition, and oxidative stability of broiler meat. Foods..

[bib0032] Liu A., Li Z., Jin X., Wu Q., Hu H., Zhang C. (2022). An encapsulated organic acid and essential oil mixture improves the intestinal health of weaned piglets by altering intestinal inflammation and antioxidative capacity. Anim.: An Open Access J. From MDPI.

[bib0033] Livak K.J., Schmittgen T.D. (2001). Analysis of relative gene expression data using real-time quantitative PCR and the 2(-Delta Delta C(T)) method. Methods (San Diego, Calif.).

[bib0034] Mead G.C. (2000). Prospects for 'competitive exclusion' treatment to control salmonellas and other foodborne pathogens in poultry. Veterinary J. (London, England: 1997).

[bib0035] Michaudel C., Sokol H. (2020). The gut microbiota at the service of immunometabolism. Cell Metab..

[bib0036] Nikoui V., Ostadhadi S., Bakhtiarian A., Abbasi-Goujani E., Habibian-Dehkordi S., Rezaei-Roshan M., Foroohandeh M., Giorgi M. (2017). The anti-inflammatory and antipyretic effects of clove oil in healthy dogs after surgery. PharmaNutr..

[bib0037] Othman S.M., Ben Naser K..M., Kanoun A.H., Salim A.A., Sherif B.M., Asheg A.A. (2022). Effect of adding clove buds powder in feed on performance and jejunum morphology in broiler chickens. Open. Vet. J..

[bib0038] Pan D., Yu Z. (2014). Intestinal microbiome of poultry and its interaction with host and diet. Gut. Microbes..

[bib0039] Pirgozliev V., Mansbridge S.C., Rose S.P., Lillehoj H.S., Bravo D. (2019). Immune modulation, growth performance, and nutrient retention in broiler chickens fed a blend of phytogenic feed additives. Poult. Sci..

[bib0040] Prates J.A.M. (2025). Improving meat quality, safety and sustainability in monogastric livestock with algae feed additives. Foods..

[bib0041] Reyer H., Zentek J., Männer K., Youssef I.M.I., Aumiller T., Weghuber J., Wimmers K., Mueller A.S. (2017). Possible molecular mechanisms by which an essential oil blend from star anise, rosemary, thyme, and oregano and saponins increase the performance and ileal protein digestibility of growing broilers. J. Agric. Food Chem..

[bib0042] Ruan D., Fan Q.L., Fouad A.M., Sun Y.Y., Huang S.S., Wu A.J., Lin C.X., Kuang Z.X., Zhang C., Jiang S.Q. (2021). Effects of dietary oregano essential oil supplementation on growth performance, intestinal antioxidative capacity, immunity, and intestinal microbiota in yellow-feathered chickens. J. Anim. Sci..

[bib0043] Sakomura N.K. (2004). Modeling energy utilization in broiler breeders, laying hens and broilers. Braz. J. Poult. Sci..

[bib0044] Seidavi, A., M. Tavakoli, F. Asroosh, C. G. Scanes, M. E. Abd El-Hack, M. A. E. Naiel, A. E. Taha, L. Aleya, K. A. El-Tarabily, A. A.-A. J. E. S. Swelum, and P. Research. 2021. Antioxidant and antimicrobial activities of phytonutrients as antibiotic substitutes in poultry feed. 29:5006-5031. 10.1007/s11356-021-17401-w 34811612

[bib0046] Sengupta A., Ghosh M., Bhattacharyya D.K. (2015). In vitro antioxidant assay of medium chain fatty acid rich rice bran oil in comparison to native rice bran oil. J. Food Sci. Technol..

[bib0047] Shang Y., Kumar S., Oakley B., Kim W.K. (2018). Chicken gut microbiota: importance and detection technology. Front. Vet. Sci..

[bib0048] Shi P., Zhang J. (2006). Contrasting modes of evolution between vertebrate sweet/umami receptor genes and bitter receptor genes. Mol. Biol. Evol..

[bib0049] Si W., Ni X., Gong J., Yu H., Tsao R., Han Y., Chambers J.R. (2009). Antimicrobial activity of essential oils and structurally related synthetic food additives towards Clostridium perfringens. J. Appl. Microbiol..

[bib0050] Sidiropoulou E., Skoufos I., Marugan-Hernandez V., Giannenas I., Bonos E., Aguiar-Martins K., Lazari D., Blake D.P., Tzora A. (2020). In vitro anticoccidial study of oregano and garlic essential oils and effects on growth performance, fecal oocyst output, and intestinal microbiota in vivo. Front. Vet. Sci..

[bib0051] Stefanello C., Rosa D.P., Dalmoro Y.K., Segatto A.L., Vieira M.S., Moraes M.L., Santin E. (2019). Protected blend of organic acids and essential oils improves growth performance, nutrient digestibility, and intestinal health of broiler chickens undergoing an intestinal challenge. Front. Vet. Sci..

[bib0052] Tian Q.Y., Piao X.S. (2019). Essential oil blend could decrease diarrhea prevalence by improving antioxidative capability for weaned pigs. Anim.: An Open Access J. From MDPI.

[bib0053] Wang H., Liang S.S., Li X.Y., Yang X.J., Long F.Y., Yang X. (2022). Effects of encapsulated essential oils and organic acids on laying performance, egg quality, intestinal morphology, barrier function, and microflora count of hens during the early laying period (vol 98, pg 6751, 2019). Poult. Sci..

[bib0054] Wu Y., Li Q., Liu J., Liu Y., Xu Y., Zhang R., Yu Y., Wang Y., Yang C. (2021). Integrating serum metabolome and gut microbiome to evaluate the benefits of lauric acid on lipopolysaccharide- challenged broilers. Front. Immunol..

[bib0055] Wu Y., Zhang H., Zhang R., Cao G., Li Q., Zhang B., Wang Y., Yang C. (2021). Serum metabolome and gut microbiome alterations in broiler chickens supplemented with lauric acid. Poult. Sci..

[bib0056] Yeom J.E., Kim S..K., Park S.Y. (2022). Regulation of the gut microbiota and inflammation by β-caryophyllene extracted from cloves in a dextran sulfate sodium-induced colitis mouse model. Molecules..

[bib0057] Youssefi M.R., Alipour R.., Fakouri Z., Shahavi M.H., Nasrabadi N.T., Tabari M.A., Crescenzo G., Zizzadoro C., Centoducati G. (2023). Dietary supplementation with eugenol nanoemulsion alleviates the negative effects of experimental coccidiosis on broiler chicken's health and growth performance. Molecules..

[bib0058] Zaazaa A., Mudalal S., Alzuheir I., Samara M., Jalboush N., Fayyad A., Petracci M. (2022). The impact of thyme and oregano essential oils dietary supplementation on broiler health, growth performance, and prevalence of growth-related breast muscle abnormalities. Anim.: An Open Access J. From MDPI.

[bib0059] Zakharzhevskaya N.B., Vanyushkina A..A., Altukhov I.A., Shavarda A.L., Butenko I.O., Rakitina D.V., Nikitina A.S., Manolov A.I., Egorova A.N., Kulikov E.E., Vishnyakov I.E., Fisunov G.Y., Govorun V.M. (2017). Outer membrane vesicles secreted by pathogenic and nonpathogenic Bacteroides fragilis represent different metabolic activities. Sci. Rep..

[bib0060] Zhang L.Y., Peng Q..Y., Liu Y.R., Ma Q.G., Zhang J.Y., Guo Y.P., Xue Z., Zhao L.H. (2021). Effects of oregano essential oil as an antibiotic growth promoter alternative on growth performance, antioxidant status, and intestinal health of broilers. Poult. Sci..

[bib0062] Zhao A., Zhang Y., Li F., Chen L., Huang X. (2023). Analysis of the antibacterial properties of compound essential oil and the main antibacterial components of unilateral essential oils. Molecules..

[bib0063] Zheng C.J., Chen Z..F., Yan X., Xiao G.S., Qiu T., Ou J.C., Cen M.Z., Li W.L., Huang Y.R., Cao Y., Zhang H.H. (2023). Effects of a combination of lauric acid monoglyceride and cinnamaldehyde on growth performance, gut morphology, and gut microbiota of yellow-feathered broilers. Poult. Sci..

[bib0064] Zhou Y., Cao D.G., Liu J., Li F.W., Han H.X., Lei Q.X., Liu W., Li D.P., Wang J. (2023). Chicken adaptive response to nutrient density: immune function change revealed by transcriptomic analysis of spleen. Front. Immunol..

[bib0065] Zhu C., Huang K., Bai Y., Feng X., Gong L., Wei C., Huang H., Zhang H. (2021). Dietary supplementation with berberine improves growth performance and modulates the composition and function of cecal microbiota in yellow-feathered broilers. Poult. Sci..

